# Adaptation of Chinese and English versions of the Ankylosing Spondylitis quality of life (ASQoL) scale for use in Singapore

**DOI:** 10.1186/s12891-017-1715-x

**Published:** 2017-08-17

**Authors:** Ying Ying Leung, Weixian Lee, Nai Lee Lui, Matthew Rouse, Stephen P. McKenna, Julian Thumboo

**Affiliations:** 1 Department of Rheumatology & Immunology, Singapore General Hospital, Singapore, Singapore; 2 Duke-NUS Medical School, Singapore, Singapore; 3 Yong Loo Lin School of Medicine, National University of Singapore, Singapore, Singapore; 4 Galen Research Ltd, Manchester, UK; 5 School of Health Sciences, University of Manchester, Manchester, UK

**Keywords:** Ankylosing spondylitis, Quality of life, Cultural adaptation

## Abstract

**Background:**

To cross-culturally adapt and validate the Singapore Chinese and Singapore English versions of the Ankylosing Spondylitis Quality of Life (ASQoL) scales.

**Methods:**

Translation of the ASQoL into Singapore Chinese and English was performed by professional and lay translation panels. Field-testing for face and content validity was performed by interviewing ten Chinese speaking and ten English speaking axial spondyloarthritis (AxSpA) patients. AxSpA patients (either Chinese or English speaking) were invited to take part in validation surveys. The Health Assessment Questionnaire (HAQ), Short Form Health Survey (SF-36), Bath Indices, and other measures of disease activity were used as comparator scales for convergent validity. A separate sample of AxSpA patients were invited to participate in a test-retest postal study, with 2 weeks between administrations.

**Results:**

The cross-sectional study included 183 patients (77% males, 82% English speaking), with a mean (SD) age of 39.4 (13.7) years. The ASQoL had excellent internal consistency (Cronbach’s alpha = 0.88), and correlated moderately with all the comparator scales. The ASQoL was able to distinguish between patients grouped by disease activity and perceived general health. The ASQoL fulfilled the Rasch model analysis for fit, reliability and unidimensionality requirements. No significant differential item functioning was noted for gender, age below or above 50 years, and language of administration. Test–retest reliability was good (*r* = 0.81).

**Conclusions:**

The ASQoL was adapted into Singapore Chinese and English language versions, and shown to be culturally relevant, valid and reliable when used with combined samples of AxSpA patients who speak either Chinese or English.

## Background

Axial spondyloarthritis (AxSpA) is a chronic inflammatory rheumatic disease affecting the spine, and to a lesser extent, the peripheral joints. It affects young adults who are economically active, causing pain and stiffness, and therefore limiting their ability to perform various activities of daily living. Over time, AxSpA can lead to structural and functional impairments and significantly impairs quality of life (QoL) [1]. QoL has been increasingly recognised as an essential outcome in chronic diseases. With the development of new treatment modalities for AxSpA, such as tumour necrosis factor (TNF) blockers, it is vital that there are accurate and reliable methods of measuring QoL in AxSpA.

The Ankylosing Spondylitis Quality of Life (ASQoL) questionnaire is a measure of QoL specific to ankylosing spondylitis (AS), and has been validated for use with SpA patients [2]. It was developed simultaneously in the United Kingdom (UK) and the Netherlands (NL) [3], and has been adapted and validated for use in numerous languages including Italian and Spanish [3–6]. Content of the ASQoL was derived from interviews with AS patients exploring the impact of the condition on their QoL. The ASQoL is based on a clear theoretical construct, the needs-based QoL model. According to the need-based model, QoL is defined as the extent to which an individual is able to meet his or her fundamental human needs. QoL is high when these needs are fulfilled and poor when they are not [7]. The model has been used as the theoretical basis in the development of patient-reported outcome measures (PROMs) for over 30 diseases, including measures for rheumatic diseases such as rheumatoid arthritis, osteoarthritis, psoriatic arthritis and systemic lupus erythematosus [8–11]. The ASQoL has been used in a number of clinical trials [12–15] and has been shown to be sensitive to change [16]. Furthermore, the ASQoL has been demonstrated to fit the Rasch model, providing evidence of its unidimensionality [3] and construct validity.

Currently, the ASQoL is not available in Singapore Chinese or Singapore English and therefore has not been available for use in this country. The aim of the present study was to develop versions of the ASQoL suitable for use in Singapore, to enable the measure to be used in multi-centre clinical trials and longitudinal studies. With a population below 7 million it is difficult to justify adapting a measure into the several different languages spoken in the country. Singapore is a multiethnic country, with 80% of Singaporeans literate in English, and 71% literate in two or more languages, PROMs available in both English and Chinese will cover 98% of the population [17]. Consequently, a decision was taken to produce Chinese and English language versions of the ASQoL and to validate them with a combined sample of Chinese and English speaking AxSpA patients. This is practical and aligned to the daily clinical practice, observational cohorts and clinical trial in the local settings.

## Methods

### Translation of the ASQoL

The ASQoL was translated into Singapore Chinese and Singapore English using the dual-panel methodology [18]. Studies have shown that the dual-panel methodology produces translations that are more acceptable to patients than the standard forward-backward methodology [19, 20]. The methodology involves conducting two independent translation panels; a bilingual panel followed by a lay panel. First, a bilingual panel produces an initial translation of the questionnaire into the target language. The bilingual panel was conducted with the presence of the original developer of ASQoL (SM), who advised on the closest meaning of the items and instructions to the original English language wording. Following this, a lay panel consisting of individuals with an average to below average educational level, assess the questionnaire items and instructions to ensure that they are written in clear, everyday language appropriate for typical patients. As the ASQoL was originally developed in English, only a lay panel was required for the Singapore English version, to ensure that the wording of item and instructions were appropriate for local patients.

### Field-testing for face and content validity

Cognitive debriefing interviews (CDIs) were conducted with Chinese speaking and English speaking patients with AxSpA to assess the relevance, acceptability and comprehensiveness of questionnaire items and instructions. AxSpA patients completed either the English or Chinese version of the ASQoL according to their primary language. Patients were chosen by the attending clinician to represent a range of disease severity, gender and age. Questionnaires were completed in the presence of a trained interviewer (YYL), who observed and made note of difficulties or hesitation in answering any items. The time of start and completion of the ASQoL were recorded. After completing the ASQoL, reasons for taking a longer time to complete specific items were elicited (eg whether the item wording was unclear, item was not relevant to the subject, etc). Patients were also asked whether all the items were relevant in assessing their QoL. With an open ended question, patients were invited to give further comments about the questionnaire and asked if any important aspects of their experience had been omitted. This method of evaluation of content validity fulfilled the recommended requirements, and has been used in the development of ASQoL [3] and other needs-based PROMs [10, 21].

### Psychometric validation

Due to the small number of SpA patients in Singapore it was decided to validate the translations with a combined sample of Singapore Chinese and Singapore English speakers. Patients fulfilling the Assessment of SpondyloArthritis international Society (ASAS) criteria for AxSpA [22] were recruited from a designated SpA outpatient clinic of a tertiary hospital. The ASAS criteria for AxSpA allow classification of patients with inflammatory back pain, other SpA features or signs of inflammation on imaging (either Magnetic Resonance Imaging or radiography); which is representative of the cases of AxSpA in daily clinical practice. Clinical features of patients with AxSpA in Singapore have been reported to be similar to those in Caucasian countries [23]. For the purpose of culturally adapting the ASQoL, we conducted a cross-sectional study with consecutive patients with AxSpA to determine the validity of ASQOL via classical theory testing (internal consistency, convergent validity and known-group validity) and Item Response Theory. We excluded non-residents (non-Singapore permanent residents or citizens), patients who could not read English or Chinese, and patients who could not give informed consent. To assess the test-retest reliability of ASQoL, a second postal survey was conducted with a separate sample of AxSpA patients who completed the ASQoL on two separate occasions, two weeks apart.

All study protocols were approved by the SingHealth Centralized Institutional Review Board (ref. CIRB 2012/498/E and 2012/696/E). Prior to their inclusion in the study, all patients provided informed consent.

### Cross-sectional study

Patients were examined by a rheumatologist and physician global assessment (PhGA) was recorded. Patients then completed a demographic questionnaire, followed by the Chinese or English ASQoL (depending on their primary language) and the comparator scales described below. All PROMs were filled in paper and pencil format in outpatient clinic.

#### ASQoL [3]

The ASQoL has 18 items, each with a dichotomous response format (True/Not true). ‘True’ responses are summed to create a total score. High scores indicate poor QoL. The original versions had excellent internal consistency (Cronbach’s apha = 0.89-0.91), test-retest reliability (*r* = 0.92 UK and 0.91 NL), and fulfilled the requirement of unidimensionality (fit to the Rasch model) [3].

#### Health Assessment Questionnaire (HAQ) [24]

This measures functional limitations in arthritic disease. It consists of 20 items covering eight categories each with a four-point scale. Total scores range from 0 ‘no disability’ to 3 ‘completely disabled’. The Chinese version of HAQ have been proven valid and reliable (test re-test reliability, *r* = 0.84) in rheumatology settings in Singapore [25].

#### Short form-36 health survey (SF-36) version 2 [26]

This is a generic health status that has been extensively validated in general population and numerous disease groups globally. The instrument consisting of 36 items which form eight subdomains (Physical Functioning, Role physical, Bodily pain, General health, Vitality, Social function, Role emotion and Mental health) and two summary scales (Physical Component Summary and Mental Component Summary). Scores for each scale are calculated by summing the items in each subdomain, which are then transformed onto a scale of 0 ‘worst health’ to 100 ‘best health’. Both English and Chinese versions of the SF-36 have been validated for use in the general population of Singapore [27]. The validity and reliability of SF-36 have been evaluated, with Cronbach’s alpha of subscales ranged from 0.88-0.90 [28].

#### The bath indices [29–31]

Patients completed the Bath Ankylosing Spondylitis Disease Activity Index (BASDAI), the Bath Ankylosing Spondylitis Functional Index (BASFI) and the Bath Ankylosing Spondylitis Global Score (BAS-G), which measure disease activity, physical functioning, and the effect of AS on the patient’s well-being in past 1 week, respectively. Items are answered using a 100 mm visual analogue scale (VAS). Scores on each of the scales range from 0 to 100, with a high score indicating higher disease activity/worse disability/greater effect on well-being.

Patient global disease activity (PGA) and perceived pain in the past week were also measured using a 100 mm VAS.

### Statistical analyses

Internal consistency was assessed using Cronbach’s alpha coefficient. Alpha measures the extent to which items in a scale are inter-related. A value of 0.7 or higher indicates adequate inter-relatedness between the items [32], and >0.90 are considered necessary for individual level use.

Convergent validity was determined by correlating scores on the new measure with those on existing PROMs. ASQoL scores were correlated with scores on the comparator measures and clinical assessments using Spearman’s rank correlation coefficients. Strong (*r* ≥ 0.7) and moderate correlation coefficients (*r* = 0.5-0.7) suggest that the scores from two PROMs are measuring related construct whereas weak correlation coefficients (r ≤ 0.3) suggest the PROMs are measuring different construct [33]. We hypothesize the ASQoL should correlate with SF-36 subscales at least moderately, and to a lesser extent with pain or global assessment indexes.

Known group validity assesses whether a scale can distinguish between groups of patients that are known to differ by some factor that would be expected to influence their scores. The factors used for the present study were physical function and disease activity (determined by HAQ and BASDAI score) and perceived general health (response to item 1 of the SF-36). Responses on the general health item were dichotomized to good (excellent/very good/good) and poor (fair/poor) for comparison. Non-parametric tests for independent samples (Mann-Whitney U test for two groups or Kruskal-Wallis One-Way Analysis of Variance for three or more groups) were employed.

### Rasch model analysis

The Rasch model analysis provides a robust evaluation of the internal construct validity of PROMs, and has been increasingly applied [34, 35]. We used WINSTEPS version 3.93.2 for Rasch model analysis and a dichotomous model was fitted. We evaluated fit to the model by a chi-square statistic, the mean-square (MNSQ) of information-weighted fit statistics (INFIT) and outlier-sensitive statistics (OUTFIT). The acceptable range of fit statistics is in the range of 0.7–1.3.A high fit statistic, > 1.3, denotes noise in the data and implies the item may not belong to the unidimensional construct. A low fit statistic, < 0.7, indicates that the item may have interdependence with another item. We examined the person separation reliability and item separation. Person separation reliability illustrates how well the index differentiates persons, while item separation provides a measure on the spread of item difficulties. An index is considered reliable if person separation reliability is >0.8.

Items of ASQoL were examined for differential item functioning (DIF) or item bias by comparing item performance for different subgroups using t-tests. Subgroups analysed included gender, age <50 years or ≥50 years, and language of administration (Chinese vs English). We reported the probability using Mantel-Haenszel statistics, a Bonferroni adjusted *p*-value of 0.0027 was considered significant.

We further examined unidimensionality via Principal Component Analysis (PCA) of the residuals [36], followed by comparison with simulation data. The absence of other meaningful patterns in the residuals were evaluated through plotting the person estimates of two subsets of positive and negative loading items (as defined by correlated at above or below 0.3 on the first factor of the PCA of the residuals).

### Test-retest reliability study

Test-retest reliability of a measure is an estimate of its reproducibility over time when no change in condition has taken place. In a separate study, the ASQoL was completed by the same patients with AxSpA on two separate occasions, 2 weeks apart. We only included patients with stable SpA, and excluded those we were expecting a change in condition, such as those having changes in medication regimen or changing exercise intervention or plan. We standardized the administrations so that the forms were completed in their rest time at home on each occasion. Two sets of questionnaires in paper and pencil format were given to patients and completed forms sent back via return envelopes. The 2 week time period was chosen because it is unlikely that the disease status will change within this short period - yet it is long enough to avoid recall bias. The test-retest reliability was assessed by correlating scores on the ASQoL on two different occasions using Spearman’s rank correlation coefficients. As data of the ASQoL are ordinal in nature, Spearman rank correlation coefficients were appropriate (intraclass correlation coefficients are also reported for information only). The Spearman’s rank correlation of >0.85 for rank correlation coefficients were considered low random measurement error and evidence of high reproducibility [37]. Other than Rasch model analysis, all analyses were performed using the SPSS version 21 statistical package.

## Results

### Translation of the ASQoL

All translation panels consisted of five participants (two males, three females). Participants in the three panels were aged from 23 to 72 years. The panels reported very few difficulties in producing translations for most of the items and instructions. In both the Chinese and English panels, the item ‘I struggle to do jobs around the house’ was interpreted as referring to housework, which in Asian culture is not usually done by men. Therefore, the panel replaced ‘jobs’ with ‘chores’ to make the item relevant to both genders. Also, the item ‘I worry about letting people down’ was changed to ‘I worry about disappointing people’. This was because, in Singapore, ‘letting people down’ may mean a person has committed a grave offence and put their family to shame. Therefore, in both language versions it was modified to convey a much milder meaning.

### Field-testing for face and content validity

Cognitive Debriefing Interviews (CDI) were conducted with ten English speaking (age range 22-55 years, 90% male, 50% married, 20% unemployed) and ten Chinese speaking (age range 24 – 60 years, 60% male, 80% married, 20% unemployed) patients with AxSpA. Overall, patients found the instrument easy to understand and relevant to their condition. Five patients highlighted areas which affected their lives that may be missed. This included restricted neck and back mobility, lower body mobility, difficult to drive long distances, impairment in work productivity. After discussion, patients agreed the above aread were assessed in item 1 (limits places can go), item 4 (struggle with chores), item 7 and 12 (tired), and item 8 (keep taking break when work). Therefore, there was no important aspects of the impact of their condition had been omitted. The patients took a mean of 3 min and 2.4 min to complete the English and Chinese versions. No changes to the questionnaires were necessary as a result of the CDIs.

### Psychometric validation

A total of 187 consecutive patients with AxSpA were recruited in the cross-sectional study, of whom 183 gave complete data. 82% and 18% completed the English and Chinese versions. Of these, 175 patients had radiographic images available. One-hundred and 45 (83%) fulfilled the New York modified criteria for sacroiliitis [38]. Among the 30 patients with radiographic features not fulfilling these criteria, 20 had active sacroiliitis on magnetic resonance imaging [22]. Seventy-seven percent of the patients were male, mean (SD) age and duration of illness were 39.5 (13.7) and 7.8 (8.9) years. Demographic and disease information are shown in Table 1. The mean (SD) ASQoL score was 4.6 (4.2). The ASQoL was noted to have a ceiling effect in 19.1% of patients, and no floor effect was noted. Table 2 shows scores on all the outcome measures.

**Table 1:** Demographic characteristics of AxSpA patients (*n* = 183) in the cross-sectional study

Age (years)		
Mean (SD)	39.5 (13.7)	
Gender	n	%
Male	141	77.0
Female	42	23.0
Marital Status		
Single	72	39.3
Married	103	56.3
Divorced/Separated	7	3.8
Widowed	1	0.5
Education		
Primary or below	6	33
Secondary	45	24.6
Post-secondary	66	36.1
Tertiary	65	35.5
missing	1	0.5
Work status		
Employed/ self employed	128	69.9
Student	19	10.4
Housekeeper	10	5.5
Retired	8	4.4
Unemployed	16	8.7
missing	2	1.1
Administered Language		
English	150	82.0
Chinese	33	18.0
Ethnicity		
Chinese	156	85.2
Malay	5	2.7
Indian	9	4.9
Others	13	7.1

**Table 2:** Descriptive statistics of the ASQoL and comparator scales (*n* = 183)

	Mean (SD)	% ceiling (Best score)	% Floor (Worse score)
ASQoL	4.6 (4.2)	19.1	0
Pain VAS (0–100)	35.0 (25.1)	3.3	0
PGA (0–100)	37.6 (24.5)	3.3	0
PhGA (1–5)	2.3 (0.9)	0	0.5
BASDAI (0–100)	33.1 (18.2)	0.5	0
BASFI (0–100)	20.5 (20.1)	12	0
BAS–G (0–100)	38.9 (21.6)	2.2	0
HAQ (0–3)	0.28 (0.39)	42.6	0
SF–36 PCS (norm-based)	42.9 (11.8)	NA	NA
SF–36 MCS (norm-based)	43.7 (12.3)	NA	NA

*VAS* visual analogue scale, *PGA* Patient Global Assessment of disease activity, *PhGA* Physician’s Global Assessment of disease activity, *BASDAI* Bath Ankylosing Spondylitis Functional Index, *BASFI* Bath Ankyosing Spondylitis Functional Index, *BAS-G* Bath Ankylosing Spondylitis Global activity assessment, *HAQ* Health Assessment Questionnaire, *SF-36* Medical Outcome Short Form-36 Physical Component Summary, *MCS* Mental Component Summary, *Norm–based* normalization to population norm with mean 50 and SD 10, *NA* not applicable

Cronbach’s alpha coefficient for the ASQoL was 0.88 indicating good internal consistency. The Cronbach’s alpha coefficient for Singapore ASQoL Chinese and English version separately were 0.93 and 0.86. Table 3 shows the Spearman’s rank correlations between ASQoL scores and scores on the comparator measures. Moderate correlations were observed between ASQoL scores and SF-36 subdomain scores and summary scores, indicating that these impairments and functional limitations influence QoL. There were moderate correlations (but lesser extent) to pain, global assessments, and physical function. This aligned with the hypothesis testing of ASQoL as a measure of QoL.

**Table 3:** Association between ASQoL scores and comparator scales

	ASQoL Spearman’s rank correlation coefficient
Pain VAS	0.50*
PGA	0.43*
PhGA	0.44*
BASDAI	0.50*
BASFI	0.44*
BAS-G	0.46*
HAQ	0.53*
SF36	
Physical Functioning	−0.60*
Role Physical	−0.60*
Bodily Pain	−0.60*
General Health	−0.59*
Vitality	−0.63*
Social Functioning	−0.64*
Role Emotional	−0.67*
Mental Health	−0.55*
Physical Component Summary	−0.60*
Mental Component Summary	−0.58*

* *p* < 0.0001

*VAS* visual analogue scale, *PGA* Patient Global Assessment of disease activity, *PhGA* Physician’s Global Assessment of disease activity, *BASDAI* Bath Ankylosing Spondylitis Functional Index, *BASFI* Bath Ankyosing Spondylitis Functional Index, *BAS-G* Bath Ankylosing Spondylitis Global activity assessment, *HAQ* Health Assessment Questionnaire, *SF-36* Short Form-36 Health Survey

**Fig. 1:**
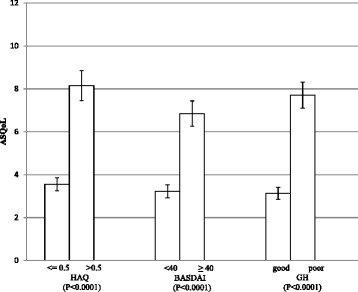
Mean ASQoL scores stratified by known-groups. HAQ = health assessment questionnaire; BASDAI = Bath ankylosing spondylitis disease activity index; GH = general health status (Good = excellent/very good/good; poor = fair/poor)

Figure 1 shows mean ASQoL scores grouped by disease status (HAQ and BASDAI scores) and perceived general health (response to item 1 of the SF-36). Significant differences were found between patients grouped by these factors, demonstrating the ability of the ASQoL to distinguish between subgroups of patients (all *p*-values <0.0001).

### Rasch model analysis

Chi-square based fit statistics are presented in Table 4. The fit of the 18 items of ASQoL were good. There were two items showing minor misfit, with MNSQ values outside the required 0.7-1.3 range. The person separation reliability was 0.87, indicating adequate reliability. The person and item separation index were 1.51 and 4.77, respectively. DIF analysis were performed for age (<50 years = 143 vs. ≥50 years =49); gender (male = 141 vs. female = 42); and language of ASQoL administered (Chinese = 33 vs. English = 150). Slight DIF was revealed with item 2 which asked about “feel like crying”, which suggests that males and females may response differently toward this item. There was no significant DIF noted by age and language (Table 4).

**Table 4:** Rasch item statistics for Singapore ASQoL

Item	Item Calibration in logits (SE)	INFIT (MNSQ)	OUTFIT (MNSQ)	DIF Probability by Mantel-Haenszel statistics (*p*)		
				Gender	Age^a^	Language^b^
#1	−0.64 (0.20)	1.14	1.25	0.4381	0.0074	0.7937
#2	0.48 (0.23)	0.99	0.89	*0.0005*	0.9200	0.5870
#3	1.56 (0.30)	1.08	0.93	0.5964	0.0037	0.6836
#4	0.63 (0.24)	0.80	0.69	0.9713	0.2586	0.9458
#5	1.09 (0.26)	0.87	0.78	0.7520	0.0476	0.9763
#6	−0.51 (0.20)	1.07	1.14	0.6056	0.8955	0.1197
#7	−0.58 (0.20)	0.95	0.87	0.0476	0.2484	0.0850
#8	−0.85 (0.20)	0.78	***0.68***	0.5995	0.9560	0.8635
#9	1.02 (0.26)	0.90	0.72	0.5301	0.5218	0.1634
#10	0.32 (0.23)	0.94	1.01	0.0631	0.9597	0.1467
#11	1.94 (0.33)	1.09	0.95	0.5933	0.6896	0.3634
#12	−1.61 (0.20)	0.86	0.74	0.1651	0.0992	0.0293
#13	−0.55 (0.20)	0.89	0.97	0.4904	0.1512	0.6042
#14	−1.69 (0.20)	***1.45***	***1.66***	0.7045	0.6147	0.0397
#15	−0.51 (0.20)	0.92	0.82	0.6857	0.2089	0.0286
#16	2.44 (0.38)	1.00	1.29	0.4703	0.0555	0.1191
#17	−1.31 (0.20)	1.13	1.12	0.4670	0.9162	0.9222
#18	−1.23 (0.20)	1.04	0.95	0.5116	0.2195	0.7969

*Bold italics =* MNSQ values outside range of 0.7 to 1.3. *Italics* = Bonferroni adjusted *p* < 0.0027 (denote concern of DIF)

*INFIT* information-weighted fit statistics, *OUTFIT* outlier-sensitive statistics, *MNSQ* mean square, *DIF* differential item functioning

^a^Age < 50 years vs. ≥50 years; ^b^Chinese vs. English

**Fig. 2:**
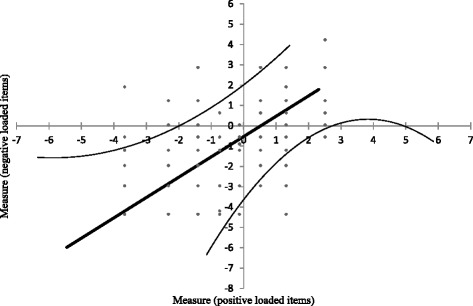
Scattered plot of person measures comparing positive and negative loading items to the first factor of the Principal component analysis of residuals

PCA of the residuals indicate that 37.2% of the variance of the residuals was explained by the Rasch model. Out of the 62.8% of the variance of residuals that were not explained, the first contrast explained 7.2%, corresponding to a eigenvalue of 2.007 (Table 5). The variance of residual explained by the Rasch model, and the Eigenvalue of the first contrast were slightly lower than the recommended 40% and 2.0. However, the percentage of variance attributed to the first residual factor compared to the Rasch factor was only 19.4%, which was within the recommended limits. We compared this result with two sets of simulation data, generated with perfect fit to the Rasch model. The simulation dataset showed similar results (Table 5). We further plotted the person estimates of two subsets of positive and negative loading items (as defined by correlated at above or below 0.3 on the first factor of the PCA of the residuals), which indicated no likelihood of a second dimension (Fig. 2). These findings suggest that when the Rasch factor was removed, there was no leftover patterns found in the residuals, thus confirming unidimensionality of the adapted ASQoL.

**Table 5:** Principal component analysis of the residuals and comparisons with simulated data

	Observed data	Simulated data 1	Simulated data 2
Total raw variance in observations	100%	100%	100%
Raw variance explained by Rasch model	37.2%	38.8%	38.4%
Raw variance explained by persons	16.1%	17.4%	16.8%
Raw variance explained by items	21.1%	21.3%	21.5%
Raw unexplained variance (total)	62.8%	61.2%	61.6%
Unexplained variance in first contrast in Eigenvalue, (%)	2.077 (7.2%)	1.67 (5.7%)	1.69 (5.8%)

### Test-retest reliability study

A separate sample of 42 AxSpA patients completed the ASQoL on two separate occasions, 2 weeks apart. Over 70% of the sample was male and the mean (SD) age was 39.1 (12.8) years. Nineteen (45.2%) patients completed the Chinese version and 23 (54.8%) the English ASQoL. There were no significant differences in patient global assessment, pain scores, and Bath Indices for these patients between two time points (all *p* values >0.19). The mean (SD) ASQoL scores at time 1 and time 2 were 4.07 (4.34) and 4.17 (4.44), respectively (*p* = 0.95). The Spearman’s rank correlation coefficient between ASQoL at time 1 and time 2 was 0.81, indicating adequate reproducibility. Limiting analysis to 36 patients who had changes of BAS-G less than the reported minimal clinically difference of <15 mm between two time points, the Spearman’s rank correlation coefficient of ASQoL at time 1 and 2 was 0.85. The intraclass correlation coefficient of ASQoL between time 1 and 2 was 0.86 (95%CI: 0.74-0.92).

## Discussion

The ASQoL was successfully translated and validated for use with Chinese and English speakers with AxSpA in Singapore. The dual-panel translation methodology has been used successfully in the adaptation of all other language versions of needs-based QoL instruments. Research has shown that this approach produces translations that are more acceptable to patients than forward-backward translation [19]. Patients who completed the CDIs found ASQoL to be relevant, applicable and easy to understand, and the ASQoL demonstrated excellent psychometric properties, with good internal consistency, convergent validity, known-group validity. The construct validity of the adapted ASQoL was supported by fulfilling the requirement of item fit, local independent of items and unidimensionality of the Rasch model. Test-retest reliability was confirmed in a separate study. These findings are comparable to those found for the original UK measure, and address the truth and feasibility filter domains of the Outcome Measure in Rheumatology (OMERACT) consensus initiative [39].

PROMs commonly used in AxSpA, such as the Bath Indices (BASDAI, BASFI, BAS-G) provide limited information on the impact of AxSpA and its treatment on the patient. QoL has been recognised by the ASAS as an important domain [40], and one of the core domains and outcome measures of rheumatic diseases in the Core Set for Longitudinal Observational Studies in Rheumatology [41]. Furthermore, maximising QoL has been recognised as the long term goal of treating AS patients in the ASAS/European League Against Rheumatism (EULAR) recommendation for management of AS [42]. The ASQoL is a disease-specific measure, and its content was derived from qualitative interviews with patients, and as a result all the items are directly relevant to AS patients. The measure was also based on a clear theatrical construct, the needs-based model of QoL. These are both essential requirements for QoL instruments in clinical research [43].

A limitation of the study was the small sample size available for the validation study. Particularly, a larger sample size is required for Rasch analysis. Recommendations suggest seven times the number of items of the PROM of interest, and >100 patients; and more than 200 patients per subgroup for proper DIF evaluation [44]. However, resource constraints did not allow us to recruit this number of AS patients, particular for the Chinese Speaking subgroup. Therefore, the small sample size for subgroups in our studies may have an impact particularly on the DIF analysis, and therefore may not be considered definite. It is also a limitation to validate the combined Chinese and English versions of ASQoL for a practical consideration. However the language of administration (English or Chinese) did not affect the psychometric properties of the ASQoL, given the low level of DIF related to language, despite the small number of patients in the Chinese subgroup. The instruments for assessing patient’s status were all relying on patient reported outcomes with the exception of PhGA, which may limit the comparison of ASQoL with a more objective assessment of disease activity. This is particularly true for the known-groups validity, which would be more robust if the "known groups" were established with a more clinical and objective standard. Nonetheless, except Ankylosing Spondylitis Disease Activity Score (ASDAS) that takes into account acute phase reactants in blood, other assessments of AxSpA at the current moment heavily rely on PROMs [12]. Besides, as the intended use of ASQoL is to measure the QoL in the patient’s perspective [45], it is relevant to use other PROMs for a comparison. It is also known that QoL is not necessarily strongly related to clinical severity. Of note is that the Bath indices used in this study were not properly culturally adapted and validated in the Singaporean languages. However, the main comparator PROMs, SF-36 and HAQ have been properly validated in our population. We acknowledge the slightly low Spearman’s coefficient (0.81) for test-retest reliability, and was improved to 0.85 when the analysis was limited to patients with more stringent criteria of “no change”. The internal consistency of the adapted ASQoL falls marginally short of the recommended (>0.90) level for individual level use. However, the Rasch model analysis supported the necessary internal consistency and structure validity of the Singapore versions of ASQoL. A sample size of at least 50 is recommended for establishing test-retest reliability [44]. The actual sample available for test-retest reliability in our study (*n* = 42) may risk producing an inaccurate estimate of this property. Finally, given the cross-sectional study design, it was not possible to evaluate the responsiveness of the new ASQoL language versions.

## Conclusion

The present study has demonstrated that the Singapore English and Chinese versions of the ASQoL are culturally relevant, valid and reliable instruments when used with combined samples of AxSpA patients who speak either Chinese or English.

## Abbreviations

AS: Ankylosing spondylitis
ASAS: Assessment of Spondylo Arthritis international Society
ASQoL: Ankylosing spondylitis quality of life scale
AxSpA: Axial spondyloarthritis
BASDAI: Bath ankylosing spondylitis disease activity index
BASFI: Bath Ankylosing spondylitis functional index
BAS-G: Bath ankylosing spondylitis global score
CDIs: Cognitive debriefing interviews
DIF: Differential item functioning
EULAR: European league against rheumatism
HAQ: Health assessment questionnaire
INFIT: Information-weighted fit statistics
MNSQ: Mean-square
NL: Netherlands
OMEARCT: Outcome measures in rheumatology
OUTFIT: Outlier-sensitive statistics
PCA: Principal component analysis
PGA: Patient global disease activity
PhGA: Physical global assessment
PROMs: Patient-reported outcome measures
QoL: Quality of life
SF-36: Medical outcome short-form 36
SpA: Spondyloarthritis
TNF: Tumour necrosis factor
UK: United Kingdom
VAS: Visual analogue scale

## References

[1] Braun J, Sieper J (2007). Ankylosing spondylitis. Lancet.

[2] Jenks K, Treharne GJ, Garcia J, Stebbings S (2010). The ankylosing spondylitis quality of life questionnaire: validation in a New Zealand cohort. Int J Rheum Dis.

[3] Doward LC, Spoorenberg A, Cook SA, Whalley D, Helliwell PS, Kay LJ (2003). Development of the ASQoL: a quality of life instrument specific to ankylosing spondylitis. Ann Rheum Dis.

[4] Doward LC, McKenna SP, Meads DM, Twiss J, Revicki D, Wong RL (2007). Translation and validation of non-English versions of the Ankylosing Spondylitis quality of life (ASQOL) questionnaire. Health Qual Life Outcomes.

[5] Pham T, van der Heijde DM, Pouchot J, Guillemin F (2010). Development and validation of the French ASQoL questionnaire. Clin Exp Rheumatol.

[6] Zhao LK, Liao ZT, Li CH, Li TW, Wu J, Lin Q (2007). Evaluation of quality of life using ASQoL questionnaire in patients with ankylosing spondylitis in a Chinese population. Rheumatol Int.

[7] McKenna SP, Doward LC, Niero M, Erdman R (2004). Development of needs-based quality of life instruments. Value Health.

[8] Whalley D, McKenna SP, de Jong Z, van der Heijde D (1997). Quality of life in rheumatoid arthritis. Br J Rheumatol.

[9] Keenan AM, McKenna SP, Doward LC, Conaghan PG, Emery P, Tennant A (2008). Development and validation of a needs-based quality of life instrument for osteoarthritis. Arthritis Rheum.

[10] McKenna SP, Doward LC, Whalley D, Tennant A, Emery P, Veale DJ (2004). Development of the PsAQoL: a quality of life instrument specific to psoriatic arthritis. Ann Rheum Dis.

[11] Doward LC, McKenna SP, Whalley D, Tennant A, Griffiths B, Emery P (2009). The development of the L-QoL: a quality-of-life instrument specific to systemic lupus erythematosus. Ann Rheum Dis.

[12] Zochling J (2011). Measures of symptoms and disease status in ankylosing spondylitis: Ankylosing Spondylitis disease activity score (ASDAS), Ankylosing Spondylitis quality of life scale (ASQoL), bath Ankylosing Spondylitis disease activity index (BASDAI), bath Ankylosing Spondylitis functional index (BASFI), bath Ankylosing Spondylitis global score (BAS-G), bath Ankylosing Spondylitis metrology index (BASMI), Dougados functional index (DFI), and health assessment questionnaire for the Spondylarthropathies (HAQ-S). Arthritis Care Res (Hoboken).

[13] Barkham N, Keen HI, Coates LC, O'Connor P, Hensor E, Fraser AD (2009). Clinical and imaging efficacy of infliximab in HLA-B27-positive patients with magnetic resonance imaging-determined early sacroiliitis. Arthritis Rheum.

[14] Barkham N, Coates LC, Keen H, Hensor E, Fraser A, Redmond A (2010). Double-blind placebo-controlled trial of etanercept in the prevention of work disability in ankylosing spondylitis. Ann Rheum Dis.

[15] Davis JC, Revicki D, van der Heijde DM, Rentz AM, Wong RL, Kupper H (2007). Health-related quality of life outcomes in patients with active ankylosing spondylitis treated with adalimumab: results from a randomized controlled study. Arthritis Rheum.

[16] Haywood KL, A MG, Jordan K, Dziedzic K, Dawes PT (2002). Disease-specific, patient-assessed measures of health outcome in ankylosing spondylitis: reliability, validity and responsiveness. Rheumatology (Oxford).

[17] Singapore Ministry of Health. Cencus of the population. 2010. [online], available at: http://www.singstat.gov.sg/publications/publications-and-papers/cop2010/census10_stat_release1. Accessed 15 Aug 2017.

[18] Swaine-Verdier A, Doward LC, Hagell P, Thorsen H, McKenna SP (2004). Adapting quality of life instruments. Value Health.

[19] McKenna SP (2011). Measuring patient-reported outcomes: moving beyond misplaced common sense to hard science. BMC Med.

[20] Hagell P, Hedin PJ, Meads DM, Nyberg L, McKenna SP (2010). Effects of method of translation of patient-reported health outcome questionnaires: a randomized study of the translation of the rheumatoid arthritis quality of life (RAQoL) instrument for Sweden. Value Health.

[21] Leung YY, Thumboo J, Rouse M, McKenna SP (2016). Adaptation of Chinese and English versions of the psoriatic arthritis quality of life (PsAQoL) scale for use in Singapore. BMC Musculoskelet Disord.

[22] Rudwaleit M, van der Heijde D, Landewe R, Listing J, Akkoc N, Brandt J (2009). The development of assessment of SpondyloArthritis international society classification criteria for axial spondyloarthritis (part II): validation and final selection. Ann Rheum Dis.

[23] Lee YX, Kwan YH, Png WY, Lim KK, Tan CS, Lui NL, et al. Association of obesity with patient-reported outcomes in patients with axial spondyloarthritis: a cross-sectional study in an urban Asian population. Clin Rheumatol. 2017; doi:https://doi.org/10.1007/s10067-017-3585-x. 10.1007/s10067-017-3585-x 28378098

[24] Fries JF, Spitz P, Kraines RG, Holman HR (1980). Measurement of patient outcome in arthritis. Arthritis Rheum.

[25] Koh ET, Seow A, Pong LY, Koh WH, Chan L, Howe HS (1998). Cross cultural adaptation and validation of the Chinese health assessment questionnaire for use in rheumatoid arthritis. J Rheumatol.

[26] Ware JE, Sherbourne CD (1992). The MOS 36-item short-form health survey (SF-36). I. Conceptual framework and item selection. Med Care.

[27] Thumboo J, Wu Y, Tai ES, Gandek B, Lee J, Ma S (2013). Reliability and validity of the English (Singapore) and Chinese (Singapore) versions of the short-form 36 version 2 in a multi-ethnic urban Asian population in Singapore. Qual Life Res.

[28] Kwan YH, Fong WW, Lui NL, Yong ST, Cheung YB, Malhotra R (2016). Validity and reliability of the short form 36 health surveys (SF-36) among patients with spondyloarthritis in Singapore. Rheumatol Int.

[29] Garrett S, Jenkinson T, Kennedy LG, Whitelock H, Gaisford P, Calin A (1994). A new approach to defining disease status in ankylosing spondylitis: the bath Ankylosing Spondylitis disease activity index. J Rheumatol.

[30] Calin A, Garrett S, Whitelock H, Kennedy LG, O'Hea J, Mallorie P (1994). A new approach to defining functional ability in ankylosing spondylitis: the development of the bath Ankylosing Spondylitis functional index. J Rheumatol.

[31] Jones SD, Steiner A, Garrett SL, Calin A (1996). The bath Ankylosing Spondylitis patient global score (BAS-G). Br J Rheumatol.

[32] Terwee CB, Bot SD, de Boer MR, van der Windt DA, Knol DL, Dekker J (2007). Quality criteria were proposed for measurement properties of health status questionnaires. J Clin Epidemiol.

[33] Hinkle D, Wiersma W, Jurs S (2003). Applied statistics for the Behavioral sciences.

[34] Tennant A, Conaghan PG (2007). The Rasch measurement model in rheumatology: what is it and why use it? When should it be applied, and what should one look for in a Rasch paper?. Arthritis Rheum.

[35] Leung YY, Png ME, Conaghan P, Tennant A (2014). A systematic literature review on the application of Rasch analysis in musculoskeletal disease -- a special interest group report of OMERACT 11. J Rheumatol.

[36] Tennant A, Pallant JF. Unidimensionality Matters! (A Tale of Two Smiths?). Rasch Measurement Transactions. 2006;20(1 Summer).

[37] Weiner E, Stewart B (1984). Assessing individuals.

[38] van der Linden S, Valkenburg HA, Cats A (1984). Evaluation of diagnostic criteria for ankylosing spondylitis. A proposal for modification of the New York criteria. Arthritis Rheum.

[39] Boers M, Brooks P, Strand CV, Tugwell P (1998). The OMERACT filter for outcome measures in rheumatology. J Rheumatol.

[40] van der Heijde D, Dougados M, Davis J, Weisman MH, Maksymowych W, Braun J (2005). ASsessment in Ankylosing Spondylitis international working group/Spondylitis Association of America recommendations for conducting clinical trials in ankylosing spondylitis. Arthritis Rheum.

[41] Wolfe F, Lassere M, van der Heijde D, Stucki G, Suarez-Almazor M, Pincus T (1999). Preliminary core set of domains and reporting requirements for longitudinal observational studies in rheumatology. J Rheumatol.

[42] Braun J, van den Berg R, Baraliakos X, Boehm H, Burgos-Vargas R, Collantes-Estevez E (2011). 2010 update of the ASAS/EULAR recommendations for the management of ankylosing spondylitis. Ann Rheum Dis.

[43] Doward LC, Meads DM, Thorsen H (2004). Requirements for quality of life instruments in clinical research. Value Health.

[44] Mokkink LB, Terwee CB, Patrick DL, Alonso J, Stratford PW, Knol DL, et al. COSMIN checklist manual. 2010. [online]. Available at: http://cosmin.nl. Accessed 15 Aug 2017.

[45] Guidance for Industry. Patient-Reported Outcome Measures: Use in Medical Product Development to Support Labeling Claims. 2009. [Online] Available at: http://www.fda.gov/downloads/Drugs/.../Guidances/UCM193282.pdf. Accessed 27 May 2017. 10.1186/1477-7525-4-79 PMC1629006 17034633

